# An Improved Mathematical Scheme for LTE-Advanced Coexistence with FM Broadcasting Service

**DOI:** 10.1371/journal.pone.0136912

**Published:** 2016-11-17

**Authors:** Zaid Ahmed Shamsan, Abdulaziz M. Al-hetar

**Affiliations:** 1 Faculty of Engineering, Al Imam Mohammad Ibn Saud Islamic University, Riyadh, Saudi Arabia; 2 Communication and Computer Dept., Faculty of Eng. and Information Tech., Taiz University, Taiz, Yemen; Universidad de Valladolid, SPAIN

## Abstract

Power spectral density (PSD) overlapping analysis is considered the surest approach to evaluate feasibility of compatibility between wireless communication systems. In this paper, a new closed-form for the Interference Signal Power Attenuation (ISPA) is mathematically derived to evaluate interference caused from Orthogonal Frequency Division Multiplexing (OFDM)-based Long Term Evolution (LTE)-Advanced into Frequency Modulation (FM) broadcasting service. In this scheme, ISPA loss due to PSD overlapping of both OFDM-based LTE-Advanced and FM broadcasting service is computed. The proposed model can estimate power attenuation loss more precisely than the Advanced Minimum Coupling Loss (A-MCL) and approximate-ISPA methods. Numerical results demonstrate that the interference power is less than that obtained using the A-MCL and approximate ISPA methods by 2.8 and 1.5 dB at the co-channel and by 5.2 and 2.2 dB at the adjacent channel with null guard band, respectively. The outperformance of this scheme over the other methods leads to more diminishing in the required physical distance between the two systems which ultimately supports efficient use of the radio frequency spectrum.

## Introduction

Long Term Evolution (LTE)-Advanced is one of the systems that proposed to reach and even surpass the requirements on International Mobile Telecommunications-Advanced (IMT-Advanced) systems, as being defined by International Telecommunication Union- Radio section (ITU-R) [1]. LTE-Advanced system (release 10 and beyond) [2] is standardized by the 3^rd^ Generation Partnership Project (3GPP) developed from Evolved Universal Terrestrial Radio Access (E-UTRA) series. LTE-Advanced uses carrier aggregation of multiple component carriers of Orthogonal Frequency Division Multiplexing (OFDM) technique to achieve high bandwidth transmission [3]. This technique is supposed to support high data rate of 100 Mbit/s and 1 Gbit/s for high and low mobility, respectively, within a wide range of frequency bands [4, 5].

In the very near future, one of the challenges that will face mobile and wireless network is spectrum shortage, where a number of large mobile wireless service providers, such as AT&T, Verizon and Sprint, state that they may not have enough spectrum to meet the mobile demands in the next few years [6]. Due to the increasing demand for more wireless bandwidth, frequency bands used by old legacy systems have been reassigned by regulatory bodies [7]. For example, the Ultra High Frequency (UHF) band, 470–862 MHz, is recently used by broadcasting services. On the other hand, in the World Radiocommunication Conferences, WRC-07 and WRC-12, sub-bands within UHF band, such as 790–862 MHz, have been allocated on co-primary operation for IMT systems which include 3G (IMT-2000) and 4G (IMT-Advanced) mobile cellular systems. The concurrent operation of the sub-band 790–862 by both analogue Frequency Modulation (FM) broadcasting service and LTE-Advanced system may create harmful inter-system interference which can impair the performance of the two systems [8]. Therefore, spectrum sharing studies should be carrying out to investigate the influence of interference from LTE-Advanced on FM broadcasting service.

In this work, we present a new interference modeling method to study the influence of LTE-Advanced interference on FM broadcasting service. We propose this method due to that the approximate-interference signal power attenuation (ISPA) method [9, 10] uses approximation calculations of LTE-Advanced interference power into FM service which causes overestimation of the interference. In contrast, this proposed method is more accurate than the existing schemes [9–12] as it takes into account realistic shapes of the interferer and victim signals. This removes a source of overestimation of the interference and allows reducing the separation distance between the two systems, which eventually supports efficient use of the radio frequency spectrum.

The conventional method used to study coexistence between dissimilar systems, minimum coupling loss (MCL) method [13], employs spectral emission mask for the transmitter to determine the maximum interference power (Imax), at each frequency offset in order to decide coexistence visibility. However, the ISPA method uses bandwidth overlap factor which is computed by multiplying of PSD of the transmitter and PSD of the receiver to investigate coexistence situation [11, 12, 14–15]. The result of the bandwidth overlapping factor is a spectral emission mask but it considers PSD of both the transmitter and the receiver [10]. In this method the size of overlapping area between the interferer and the victim plays an important role in estimating the power loss due to overlapping. In addition, the shape of the receiver PSD determines the size of overlapping area between the two systems. For example, if there are receiver-A with rectangular PSD and receiver-B with triangular PSD and both of them use the same bandwidth, then the size of overlapping area with receiver-A will be larger than that of receiver-B, due to that the area is obtained according to the shape of the receiver PSD. Specifically, when the size of overlapping area between two systems is large the receiver will suffer from high interference power and this is maximum when the carrier frequency of the two systems is identical because the interference power loss is small, and vice versa.

The main contributions of this paper are listed as follows. (i) We model the inter-system interference between OFDM-based LTE-Advanced and FM broadcast systems depending on realistic power spectral density (PSD) shapes of the interferer and victim systems, (ii) we derive a new closed-form for the interfering signal power attenuation (ISPA) which can more exact evaluate interference caused by LTE-Advanced system into FM service than other schemes, and (iii) we investigate, through extensive analysis, performance results of the proposed scheme against previously methods, including co-channel and adjacent channel scenarios, and propose some mechanisms to improve coexistence between the two systems.

The rest of this paper is organized as follows. We present related work in the 2^nd^ section. In the third section, the proposed ISPA method is mathematically derived in a closed-form. The 4^th^ section presents the simulation assumption for spectrum sharing study. Numerical results, discussion and analysis are addressed in the 5^th^ section. Conclusion remarks are finally shown in the last section.

## Related Work

In general, interference in wireless communication can be broadly classified into co-channel (operating in the same frequency channel) and adjacent channel [16]. In addition, wireless system coexistence issue, due to interference, can be within one wireless system (intra-system interference) [17–19] or between different systems (inter-system interference) [16]. There are several statistical and deterministic approaches have been proposed to study and analyse coexistence and spectrum sharing between dissimilar wireless communication systems. The most well-known statistical approach for spectrum sharing studies is the Monte Carlo method which is being now used by European Communications Office to develop the Spectrum Engineering Advanced Monte Carlo Analysis Tool (SEAMCAT) software [13]. Whereas, the main common deterministic method used for spectrum sharing investigation purpose is the Minimum Coupling Loss (MCL) [13].

Due to MCL approach limitation, another method called Advanced-MCL (A-MCL) approach has been proposed by [11, 12]. However, A-MCL is not suitable for studying the interference from OFDM-based systems into Frequency Modulation (FM) broadcasting service that has a triangular power spectral density (PSD) shape [9, 10, 20, 21]. Therefore, the approximate-interference signal power attenuation (ISPA) method is derived by [9, 10] to evaluate interference from IMT-Advanced systems to FM broadcasting. Authors in [9, 10] have calculated interference power attenuation loss as an approximation value by assuming the summation of an equal three rectangular overlapping areas of PSD minus the non overlapping ones, which does not really express the exact bandwidth overlapping ratio.

However, In this paper and based on [9, 10], we propose a new closed-form expression which can evaluate exact ISPA loss due to overlapping of PSD of OFDM-based LTE-Advanced systems (as an interferer) and PSD of FM broadcasting service (as a victim). In this approach, the interference power loss or overlapping bandwidth ratio is computed by an integration of the combined received OFDM PSD over the real shape bandwidth (triangular) of receiver, and then the result of integration is divided by the full amount of power in the transmitter PSD. The process we use to calculate the integration in this paper, instead of assuming equal rectangular overlapping areas (approximate method) in [9, 10], determines the real resulting interference area which is more less than that obtained by [9, 10]. This leads to the fact that the interference power loss is higher which means less effect of LTE-Advanced interference. Therefore, the resulting closed-form derived by the proposed scheme can more exact evaluate interference caused by LTE-Advanced system into FM broadcasting service than other methods.

## The Proposed ISPA Method

For spectrum sharing analysis purpose, evaluation of coexistence feasibility needs to use one of standard protection criteria or thresholds that have been suggested by international regulator bodies, such as the ITU-R, Institute of Electrical and Electronics Engineers (IEEE) or European Conference of Postal and Telecommunications Administrations (CEPT), etc. The maximum allowable interference power (*I*_*max*_) at the antenna of an interfered victim receiver is proposed in this paper as a standard criteria [20, 22–24]. Therefore, once the interferer system (LTE-Advanced) operates concurrently with the intefered system (FM broadcasting receiver) the essential minimum value of attenuation loss (*L*_*ma*_), in dB, can be described by:

$$L_{ma}=P_{t}+G_{t}+G_{r}+L_{a}-I_{max}$$ (1)

where *P*_*t*_(dBW) is the transmit power of the LTE-Advanced system in the reference bandwidth, *G*_*t*_(dBi) is the LTE-Advanced transmitter antenna gain, and *G*_*r*_(dBi) is the victim FM broadcasting receiver antenna gain. The interfering signal power attenuation (ISPA) is denoted by *L*_*a*_.

**Fig 1:**
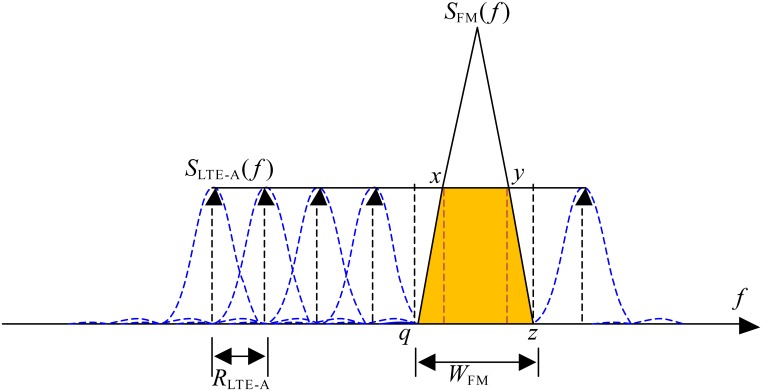
The spectrum sharing model due to overlapping of PSD of OFDM-based LTE-Advanced system and FM broadcasting system.

The resulting ISPA loss for the LTE-Advanced interfering system can be derived through a PSD analysis of the OFDM signals in this approach. The PSD of the analogue FM broadcasting system has a shape that is a triangle as Eq (2) shows.

$$S_{FM}\left(f\right)=\frac{2P_{FM}}{W_{FM}}\text{tri}\left(\frac{2f}{W_{FM}}\right)$$ (2)

where tri (∙) represents the FM broadcasting triangular function, *P*_*FM*_ is the transmit power and *W*_*FM*_ is bandwidth of FM broadcasting system. Assuming that OFDM-based LTE-Advanced system has *M* subcarriers, the PSD of the OFDM signal is a rectangular pulse shape and can be expressed as [11, 12].

$$S_{LTE-A}\left(f\right)=\overset{M-1}{\underset{i=0}{\sum }}\frac{P_{LTE-A}}{R_{LTE-A}}{\text{sinc}}^{2}\left(\frac{f}{R_{LTE-A}}-i\right)$$ (3)

where each OFDM subcarrier has a power of *P*_*LTE*−*A*_, and *R*_*LTE*−*A*_ is the spacing between each two subcarriers. The function sinc(*x*) is equivalent to sin*(πx)/πx*. Because that LTE-Advanced system will support, at least, high data rates (100 Mbps-1Gbps), these rates have need of high bandwidth. This again means that bandwidth of LTE-Advanced system (*W*_*LTE-A*_) will be often (if not always) greater than that of FM Broadcasting service (*W*_*FM*_). Therefore, the colored area in Fig 1 shows the spectrum overlapping of the PSD of OFDM-based LTE-Advanced and PSD of FM broadcasting systems, where the *W*_*LTE_A*_, is assumed to be always greater than *W*_*FM*_.

The ISPA can be calculated by an integration of the combined PSD that is received over the receiver’s bandwidth. Then, the result of integration is divided by the total power in the PSD of transmitter. Thus, the three colored overlapping areas (*q*-*x*, *x*-*y* and *y*-*z*) shown in Fig 1 can be expressed as Eq (4).

$$L_{a}=10\;\text{log}\left(\frac{\int_{\text{q}}^{\text{x}}A_{1}\left(f\right)df+\int_{\text{x}}^{\text{y}}A_{2}\left(f\right)df+\int_{\text{y}}^{\text{z}}A_{3}\left(f\right)df}{P_{t}}\right)$$ (4)

where *A*_1_(*f*), *A*_2_(*f*), and *A*_3_(*f*) are the overlapping areas between PSD for LTE-A and FM systems. These areas are obtaining by integrating the combined PSD that is received over the FM receiver’s bandwidth. The area of *A*_1_(*f*) can be expressed by multiplying the straight line *qx* and PSD of LTE-A denoted by *S*_*LTE*−*A*_(*f*) as follows:

$$A_{1}\left(f\right)=C_{qx}S_{LTE-A}\left(f\right)$$

where *C*_*qx*_ = *C*_1_*f* + *C*_2_ is the straight line equation with *C*_1_ and *C*_2_ parameters, and *S*_*LTE*−*A*_(*f*) is defined in Eq (3). So, *A*_1_(*f*) can be written as

$$A_{1}\left(f\right)=\left(C_{1}f+C_{2}\right)S_{LTE-A}\left(f\right).$$

To calculate the parameters *C*_1_ and *C*_2_, we must determine the corresponding values for the points *q*, *x*, *y*, and *z* in terms of FM bandwidth, *W*_*FM*_. From Fig 1, it is clear that the points *q* and *z* are equal to $\frac{-W_{FM}}{2}$ and $\frac{W_{FM}}{2}$ from the FM center bandwidth, respectively. Whereas, the points *x* and *y*, in reality, depend on the height of the triangle which in turn depends on the power of the FM transmission and the bandwidth *W*_*FM*_ in addition to the height of the LTE-A spectrum, i.e., the PSD *P*_*LTE*−*A*_. Due to the fact that the FM TV transmitter power, *P*_*FM*_, is much higher than that of the LTE-A system, *P*_*LTE*−*A*_, based on the Federal Communications Commission (FCC) and ITU-R regulations in the UHF bands, the FM spectrum is expected to be taller than the LTE-A spectrum, i.e., *P*_*FM*_ > *P*_*LTE*−*A*_, as depicted in Fig 1. For simplification of mathematical analysis, the points *x* and *y* are assumed to be mid-way between the center of the FM spectrum and its two edges, i.e., they are corresponding to $\frac{-W_{FM}}{4}$ and $\frac{W_{FM}}{4}$ respectively, relative to center of the FM spectrum.

Consequently, the parameter *C*_1_ (or the slop) can be calculated as follows:

$$C_{1}=\frac{P_{LTE-A}}{\frac{W_{FM}}{4}}=\frac{4P_{LTE-A}}{W_{FM}}.$$

To obtain *C*_2_, we use the equation above: *C*_*qx*_ = *C*_1_*f* + *C*_2_ = 0, thus

$$C_{2}=-C_{1}f=-C_{1}\left(f_{c}-\frac{W_{FM}}{2}\right)=C_{1}\left(\frac{W_{FM}}{2}-f_{c}\right)$$

$$=\frac{4P_{LTE-A}}{W_{FM}}\left(\frac{W_{FM}}{2}-f_{c}\right).$$

Similarly, *A*_2_(*f*) and *A*_3_(*f*) are obtained in the same way, as follows:

$$A_{2}\left(f\right)=C_{xy}S_{LTE-A}\left(f\right)=P_{LTE-A}S_{LTE-A}\left(f\right)$$

where *C*_*xy*_ = *P*_*LTE*−*A*_

$$A_{3}\left(f\right)=C_{yz}S_{LTE-A}\left(f\right)$$

$$A_{3}\left(f\right)=\left(-C_{1}f+C_{3}\right)S_{LTE-A}\left(f\right).$$

To obtain *C*_3_, we use the equation above: *C*_*yz*_ = −*C*_1_*f* + *C*_3_ = 0, thus

$$C_{3}=C_{1}f=C_{1}\left(f_{c}+\frac{W_{FM}}{2}\right)$$

where *fc* is the center frequency of the victim receiver. The points *q*, *x*, *y*, and *z* in Fig 1 determine the overlapping area between OFDM’s PSD and FM’s PSD, and these points represent *f*_*c*_−(*W*_*FM*_/2), *f*_*c*_−(*W*_*FM*_/4), *f*_*c*_+(*W*_*FM*_/4) and *f*_*c*_+(*W*_*FM*_/2), respectively.

The Eq (4) is further simplified using trigonometric functions powers and MacLaurine series in [25], so we can find that *u* = *π*(*f*/*R*_*LTE*−*A*_ − *i*), *h*_1_ = (*f*_*c*_/*R*_*LTE*−*A*_)(*W*_*FM*_/2*R*_*LTE*−*A*_), *h*_2_ = (*f*_*c*_/*R*_*LTE*−*A*_)(*W*_*FM*_/4*R*_*LTE*−*A*_), *h*_3_ = (*f*_*c*_/*R*_*LTE*−*A*_) + (*W*_*FM*_/4*R*_*LTE*−*A*_), and *h*_4_ = (*f*_*c*_/*R*_*LTE*−*A*_) + (*W*_*FM*_/2*R*_*LTE*−*A*_).

From (2)–(4)

$$\overset{\text{x}}{\underset{\text{q}}{\int }}A_{1}\left(f\right)df=\overset{\text{x}}{\underset{\text{q}}{\int }}\left(C_{1}f+C_{2}\right)\overset{M-1}{\underset{i=0}{\sum }}\frac{P_{LTE-A}}{\pi }\frac{{\text{sinc}}^{2}\left[\pi \left(f/R_{LTE-A}-i\right)\right]}{\pi {\left(f/R_{LTE-A}-i\right)}^{2}}df$$

$$=\overset{M-1}{\underset{i=0}{\sum }}\frac{P_{LTE-A}}{\pi }\left[C_{1}\frac{R_{LTE-A}}{\pi }\overset{\text{x}}{\underset{\text{q}}{\int }}\frac{{\text{sin}}^{2}u}{u}du+\left(C_{1}iR_{LTE-A}+C_{2}\right)\overset{\text{x}}{\underset{\text{q}}{\int }}\frac{{\text{sin}}^{2}u}{u^{2}}du\right].$$ (5)

Then, by using trigonometric functions, sin^2^(*x*) = (1-cos(2*x*))/2, the integral in Eq (5) can be evaluated using Eqs (6) and (7), where

$$\overset{b}{\underset{a}{\int }}\frac{{\text{sin}}^{2}u}{u}du=\frac{1}{2}\overset{b}{\underset{a}{\int }}\frac{1-\text{cos}2u}{u}du=\frac{1}{2}\left[\overset{b}{\underset{a}{\int }}\frac{1}{u}du-\overset{b}{\underset{a}{\int }}\frac{\text{cos}2u}{u}du\right]$$ (6)

$$\overset{b}{\underset{a}{\int }}\frac{{\text{sin}}^{2}u}{u^{2}}du=\frac{1}{2}\overset{b}{\underset{a}{\int }}\frac{1-\text{cos}2u}{u^{2}}du=\frac{1}{2}\left[\overset{b}{\underset{a}{\int }}\frac{1}{u^{2}}du-\overset{b}{\underset{a}{\int }}\frac{\text{cos}2u}{u^{2}}du\right].$$ (7)

Using $\overset{b}{\underset{a}{\int }}\frac{1}{u}du=\text{ln}\left(b\right)-\text{ln}\left(a\right),\;\overset{b}{\underset{a}{\int }}\frac{1}{u^{2}}du=\frac{1}{a}-\frac{1}{b},$ and MacLaurinew series in [25]

$$\overset{b}{\underset{a}{\int }}\frac{\text{cos}2u}{u}du=\text{ln}\left(b\right)+\overset{\infty }{\underset{k=1}{\sum }}{\left(-1\right)}^{k}\frac{{\left(2\text{b}\right)}^{2k}}{2k\left(2k\right)!}-\left[\text{ln}\left(b\right)+\overset{\infty }{\underset{k=1}{\sum }}{\left(-1\right)}^{k}\frac{{\left(2\text{a}\right)}^{2k}}{2k\left(2k\right)!}\right]$$ (8)

$$\overset{b}{\underset{a}{\int }}\frac{\text{cos}2u}{u^{2}}du=\frac{\text{cos}2a}{a}-\frac{\text{cos}2b}{b}-2\overset{b}{\underset{a}{\int }}\frac{\text{sin}2u}{u^{2}}du$$ (9)

$$\overset{b}{\underset{a}{\int }}\frac{\text{sin}2u}{u^{2}}du=\;\overset{\infty }{\underset{k=1}{\sum }}\frac{{\left(-1\right)}^{k-1}}{\left(2k-1\right)\left(2k-1\right)!}\times \left[{\left(2b\right)}^{2k-1}-\;{\left(2a\right)}^{2k-1}\right].$$ (10)

Therefore, employing Eqs (8), (9) and (10), both Eqs (6) and (7) can be written as Eqs (11) and (12), respectively,

$$\overset{b}{\underset{a}{\int }}\frac{{\text{sin}}^{2}u}{u}du=\frac{1}{2}\overset{\infty }{\underset{k=1}{\sum }}{\left(-1\right)}^{k}\frac{{\left(2a\right)}^{2k}-{\left(2\text{b}\right)}^{2k}}{2k\left(2k\right)!}$$ (11)

$$\overset{b}{\underset{a}{\int }}\frac{{\text{sin}}^{2}u}{u^{2}}du=\frac{1}{2}\left[\frac{1}{a}-\frac{1}{b}-\frac{\text{cos}2a}{a}+\frac{\text{cos}2b}{b}+2\overset{\infty }{\underset{k=1}{\sum }}\frac{{\left(-1\right)}^{k-1}\left[{\left(2b\right)}^{2k-1}-\;{\left(2a\right)}^{2k-1}\right]}{\left(2k-1\right)\left(2k-1\right)!}\right].$$ (12)

Employing Eqs (11) and (12), Eq (5) can be represented as Eq (13)

$$\begin{array}{l} \int_{\text{q}}^{\text{x}}A_{1}\left(f\right)=\overset{M-1}{\underset{i=0}{\sum }}\frac{P_{LTE-A}}{2\pi }\{\frac{C_{1\;}R_{LTE-A}}{\pi }\left[\overset{\infty }{\underset{k=1}{\sum }}\frac{{\left(-1\right)}^{k}\left[{\left(2\pi \left(h_{1}-i\right)\right)}^{2k}-{\left(2\pi \left(h_{2}-i\right)\right)}^{2k}\right]}{\left(2k\right)\left(2k\right)!}\right]\\ +\left[C_{1}R_{LTE-A}i+C_{2}\right][\frac{1}{\pi \left(h_{1}-i\right)}-\frac{1}{\pi \left(h_{2}-i\right)}-\frac{\text{cos}2\pi \left(h_{1}-i\right)}{\pi \left(h_{1}-i\right)}+\frac{\text{cos}2\pi \left(h_{2}-i\right)}{\pi \left(h_{2}-i\right)}\\ +2\overset{\infty }{\underset{k=1}{\sum }}\frac{{\left(-1\right)}^{k-1}\left[{\left(2\pi \left(h_{2}-i\right)\right)}^{2k-1}-{\left(2\pi \left(h_{1}-i\right)\right)}^{2k-1}\right]}{\left(2k-1\right)\left(2k-1\right)!}]\}=Y_{1} \end{array}$$ (13)

Similarly, the integration for *A*_2_(*f*)*df* and *A*_3_(*f*)*df* can be expressed by Eqs (14) and (15).

$$\begin{array}{l} \int_{\text{x}}^{\text{y}}A_{2}\left(f\right)df\\ =\overset{M-1}{\underset{i=0}{\sum }}\left(P_{LTE-A}\right)\frac{P_{LTE-A}}{2\pi }\{[\frac{1}{\pi \left(h_{2}-i\right)}-\frac{1}{\pi \left(h_{3}-i\right)}-\frac{\text{cos}2\pi \left(h_{2}-i\right)}{\pi \left(h_{2}-i\right)}+\frac{\text{cos}2\pi \left(h_{3}-i\right)}{\pi \left(h_{3}-i\right)}\\ +2\overset{\infty }{\underset{k=1}{\sum }}\frac{{\left(-1\right)}^{k-1}\left[{\left(2\pi \left(h_{3}-i\right)\right)}^{2k-1}-{\left(2\pi \left(h_{2}-i\right)\right)}^{2k-1}\right]}{\left(2k-1\right)\left(2k-1\right)!}]\}=Y_{2} \end{array}$$ (14)

$$\begin{array}{l} \int_{y}^{z}A_{3}\left(f\right)df=\overset{M-1}{\underset{i=0}{\sum }}\frac{P_{LTE-A}}{2\pi }\{\frac{-C_{1}R_{LTE-A}}{\pi }\left[\overset{\infty }{\underset{k=1}{\sum }}\frac{{\left(-1\right)}^{k}\left[{\left(2\pi \left(h_{3}-i\right)\right)}^{2k}-{\left(2\pi \left(h_{4}-i\right)\right)}^{2k}\right]}{\left(2k\right)\left(2k\right)!}\right]+\left[C_{3}-C_{1}R_{LTE-A}i\right][\frac{1}{\pi \left(h_{3}-i\right)}-\frac{1}{\pi \left(h_{4}-i\right)}\\ -\frac{\text{cos}2\pi \left(h_{3}-i\right)}{\pi \left(h_{3}-i\right)}+\frac{\text{cos}2\pi \left(h_{4}-i\right)}{\pi \left(h_{4}-i\right)}+2\sum_{k=1}^{\infty }\frac{{\left(-1\right)}^{k-1}\left[{\left(2\pi \left(h_{4}-i\right)\right)}^{2k-1}-{\left(2\pi \left(h_{3}-i\right)\right)}^{2k-1}\right]}{\left(2k-1\right)\left(2k-1\right)!}]\}=Y_{3} \end{array}$$ (15)

Therefore, the ISPA loss, in dB, can be written as Eq (16).

$$L_{a}=10\text{log}\left[\frac{Y_{1}+Y_{2}+Y_{3}}{P_{t}}\right].$$ (16)

The infinite series terms in Eqs (13), (14) and (15) are convergent and the Test Ratio method can be used to verify the convergence as shown in the appendix.

## Assumptions and Coexistence Parameters

According to [9, 10], it is assumed that LTE-Advanced system employs cellular OFDM/OFDMA technology and uses the duplexing mode, TDD. For FM TV receiver, the maximum allowable interference power (*I*_*max*_) is -146 dBW/6 MHz [9, 10] which is calculated based on the interference to noise ratio (*I*/*N*), *I*/*N* = −10 dB for co-primary spectrum sharing operation [22–24]. Where, the noise floor for FM TV receiver is -106 dBm [26], thus *I* = *N* +(−10) = −106−10 = −116 dBm. For consistency, this unit should be expressed in dBW instead of dBm according to the formula: Power (dBW) = Power (dBm) − 30 dB = −146 dBW/6 MHz. This value has been also considered in [9, 10]. LTE-Advanced system may employ base stations with a sectored antennas assumed to radiate at a center frequency of 800 MHz, whereas FM TV receiver uses a directional antenna [27]. In link budget, for most studied scenarios radiation pattern of antennas is not considered excepting for the maximum gain, and they are assumed to be omnidirectional antennas so that the worst condition scenarios can be investigated. However, LTE-Advanced antenna pattern [28] is considered in one case. For spectrum sharing situation, as approved by ITU-R [29], channel fading effect of the signal is assumed to be consisted of free space loss (*L*_*fs*_) plus the effect of clutter loss (*A*_*h*_) model. This propagation model has been considered in many coexistence and spectrum sharing studies according to ITU-R [29]. Additionally, losses of 20 and 50 dB created by discrimination of antennas, (*A*_*D*_), are taken into account. The minimum value of attenuation loss, denoted by *L*_*ma*_, as in Eq (1), can be translated into a geographical separation, that is the minimum separation distance, *d*, by manipulating the total propagation loss, *L*_*t*_, as follows:

$$L_{ma}=P_{t}+G_{t}+G_{r}+L_{a}-I_{max}=L_{t}$$ (17)

where the total propagation loss is

$$L_{t}=L_{fs}+A_{h}+A_{D}$$ (18)

$$L_{fs}=32.5+20\;\text{log}\left(f=800\;\text{MHz}\right)+20\;\text{log}\left(d\right)$$ (19)

thus

$$L_{ma}=P_{t}+G_{t}+G_{r}+L_{a}-I_{max}-\left(32.5+20\;\text{log}\left(800\right)+20\;\text{log}\left(d\right)+A_{h}+A_{D}\right)$$ (20)

Therefore, the minimum separation distance can be expressed as follows

$$d=10^{\left(P_{t}+G_{t}+G_{r}+L_{a}-I_{max}-\left(32.5+20\;\text{log}\left(800\right)+A_{h}+A_{D}\right)/20\right)}$$ (21)

channel fading effect of the signal is assumed to be consisted of free space loss plus the effect of clutter loss model [30]. Additionally, losses of 20 and 50 dB created by discrimination of antennas are taken into account. Due to that IMT-Advanced systems support high data rates, high bandwidth is necessary. Therefore, in Fig 1 the bandwidth of OFDM-based LTE-Advanced, *W*_*LTE_A*_, is assumed to be always greater than that of FM television, *W*_*FM*_. The spectrum sharing parameters for OFDM-based LTE-Advanced and FM TV receiver are displayed in Table 1.

**Table 1:** The parameters of coexistence between OFDM-based LTE-Advanced and FM Broadcasting [9, 10, 27–31].

Parameter	Value
Carrier frequency (*f*_*c*_)	0.8 GHz
LTE-Advanced base station transmitted power (*P*_*t*_)	0–15 dBW
OFDM subcarrier frequency spacing (*R*_*LTE*−*A*_)	10.24 kHz
No. of OFDM subcarriers (*M*)	1024
OFDM subcarrier power (*P*_*LTE*−*A*_ *= P*_*t*_*/M*)	(*P*_*t*_/1024) W
OFDM channel bandwidth (*W*_*LTE-A*_ *= R*_*LTE*−*A*_×*M*)	10.48576 MHz
FM receiver bandwidth (*W*_*FM*_)	6 MHz
LTE-Advanced transmitter antenna gain (*G*_*t*_)	14.5 dBi
LTE-Advanced transmitter antenna height	30 m
FM receiver antenna gain (*G*_*r*_)	12 dBi
FM receiver antenna height	10 m
FM receiver noise floor (*N*)	-106 dBm
*I/N* ratio at FM broadcasting receiver	-10 dB
Maximum permissible interference power (co-primary operation, *I/N =* -10 dB)	-146 (dBW/6 MHz)
Clutter loss (*A*_*h*_)	20 dB

## Numerical Results and Coexisting Analysis

**Fig 2:**
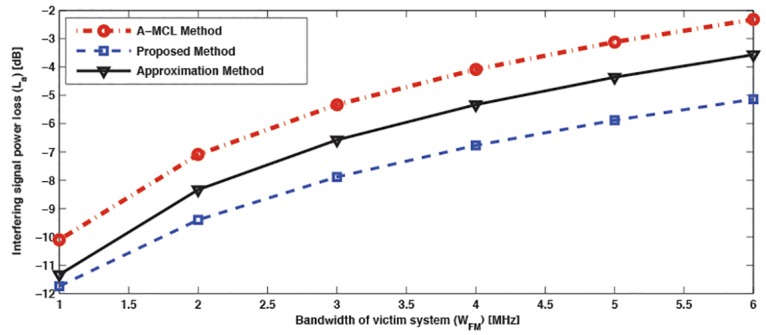
The interfering power loss versus different bandwidth of victim system at co-channel for the proposed ISPA, approximate ISPA, and A-MCL methods.

In this study, the results are based on assumption presented in the previous section. Fig 2 shows the outperformance of the proposed scheme over other methods, where ISPA loss is depicted for the proposed scheme and both A-MCL and approximate-ISPA methods at co-channel frequency, for a range of channel bandwidths (1–6 MHz) of the interfered system. It can be noted that the loss in interference power by the proposed ISPA scheme at bandwidth of TV receiver of 6 MHz is 5.1 dB and it is higher than that obtained by the A-MCL and approximate methods which they achieve 2.3 and 3.6 dB, accordingly. Additionally, we can clearly see that as bandwidth of the victim system decreases the resulting loss goes up to higher levels. It is worth noting that the proposed scheme suffers from the highest losses. This scenario is really true because that as bandwidth of the recevier increases the overlapping area with interferer system also increases and this leads to make interference power higher. Whereas, if the bandwidth of the receiver is small the overlapping bandwidth ratio will be also small and thus the level of interference power comes to the victim system with lower value.

**Fig 3:**
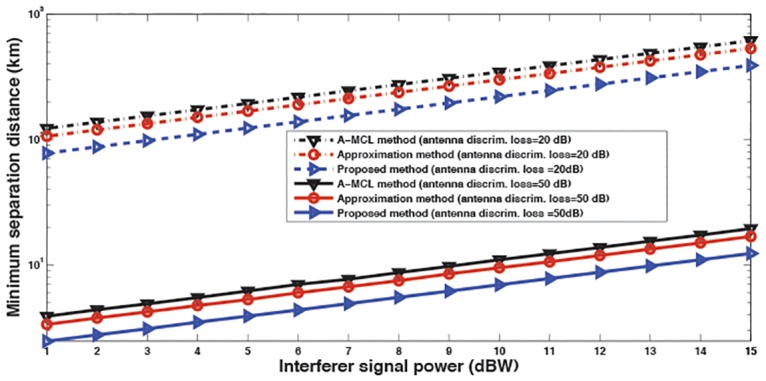
The minimum distances required by the proposed ISPA, approximation and A-MCL methods at co-channel frequency.

As an advantage for the proposed scheme, Fig 3 displays the required minimum physical distance versus transmit power of an LTE-Advanced system. Fig 3 shows that the physical separation is more less using the proposed ISPA scheme. The range of the necessary distance of both the A-MCL and proposed methods is roughly 29.6 to 148 km and 0.9 to 4.7 km for interferer power of 0 to 15 dBW and discrimination loss of 20 and 50 dB, in that order.

As a result, the proposed scheme appears more positive especially when coping with higher interferer power. These results are unsurprising considering that the overlapping area between PSD of both FM and OFDM-based LTE-Advanced of the present model is less than that in the case of A-MCL method. Additionally, Fig 3 shows that by using the proposed method, there is a linear increasing in interference power received by FM system; simultaneously this interference power is smaller when it is compared to the A-MCL and approximation approaches. Consequently, the proposed method can determine more exact interference power (more interference power loss level) that leads to more reducing in the required distance between the two systems, which in turn supports efficient use of the spectrum resource.

In spectrum sharing it is also significant to investigate coexistence feasibility at different adjacent frequencies or frequency shifts (Δ*f*) which undoubtedly have different effective power levels on coexistence situation.

**Fig 4:**
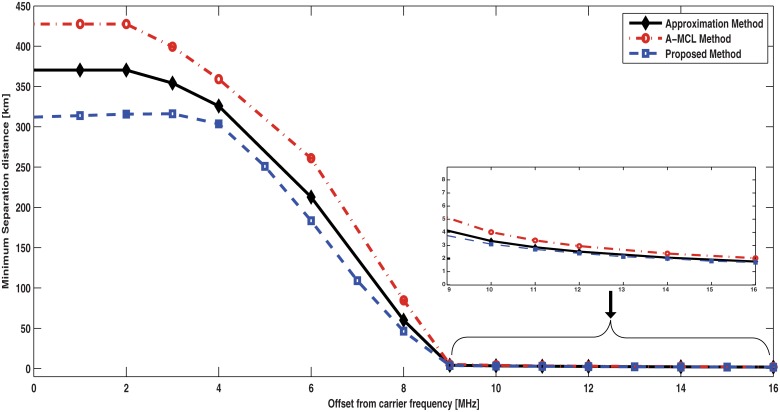
The minimum separation distances versus different carrier offsets by the proposed ISPA, approximate ISPA, and the A-MCL methods.

The attenuation loss of interfering power at different spectral (Δ*f*) from the carrier frequency, which is created due to bandwidth overlapping between the LTE-Advanced’s PSD and TV FM’s PDF, can be obtained using Eq (16). In Fig 4, frequency values between Δ*f* = 0 MHz (co-channel frequency) and Δ*f* = 15 MHz and using interfering power of 13 dBW are employed to investigate the required physical distance between the two systems. It is shown, in Fig 4, that the ISPA loss for the proposed scheme looks like the positive side of spectrum emission mask with more restriction than both the A-MCL and approximate-ISPA methods in terms of interfering signal especially when Δ*f* has greater values than 5 MHz. Moreover, it is clearly seen in Fig 4 that, because attenuation loss of power level is dramatically increased as the operation carrier goes away from the center frequency, the minimum distance is more reduced as Δ*f* becomes greater.

As seen in Fig 4, the required distance obtained by the proposed method at co-channel (Δ*f =* 0 MHz) is 312 km whereas it equals 427 and 370 km for the A-MCL and the approximate methods, correspondingly. On the other hand, the necessary distance becomes smaller at adjacent frequencies, i.e. Δ*f* > 0 MHz. For example, at Δ*f =* 8 MHz (it matches to a guard band of 0 MHz between the two systems) the minimum distance is 46, 60 and 84 km for the proposed method, approximate-method, and A-MCL method, respectively. Moreover, the separation distance becomes more less if a guard band is inserted between the two systems, for example, the distance reduces to be 2.03, 1.77 and 1.7 km when 8 MHz is added as a protection band between LTE-Advanced and FM TV systems for the A-MCL method, approximate-method and proposed method, in that order. From these results, we can see that the minimum distance is inversely proportional to the size of the protection band. These findings are predicted because the computed bandwidth overlapping ratio by the proposed scheme is smaller and more exact computed compared to other methods, which leads to a bigger isolation and eventually shorter separation distance.

Furthermore, in Fig 4, it can be seen that when offset is 9 MHz or higher, all these methods have very similar performance. In fact, the bandwidth overlapping factor has high effect when the carrier frequency of the interferer system is more close to the carrier frequency of the victim system (i.e., small frequency offset) and effect of this factor is higher when the offset is zero Hz. Whereas, when the carrier offset goes far away from the carrier frequency there is no overlapping, so we can see that all these methods have very similar performance at 9 MHz or more because bandwidth overlapping factor effect starts to be negligible at 9 MHz or higher, where every one of these methods has its technique to consider overlapping area when there is overlapping. However, when there is no overlapping or it is very small all methods have similar behavior.

**Fig 5:**
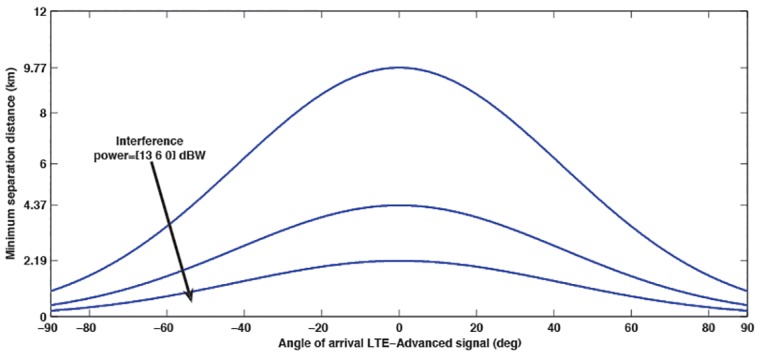
The minimum distances required due to different arrival angle of LTE-Advanced and different interference powers.

In Fig 5, the proposed base station sectored antenna of LTE- Advanced in [28] is considered with an additional loss of 50 dB and different interference power, such that the separation distance is controlled by the angle of arrival of LTE-Advanced antenna pattern. It clearly shows that for an arrival angle *θ* = 0°, the antenna discrimination loss is 0 dB, so the results are pessimistic. Moreover, It should also be noted that the distance between OFDM-based LTE-Advanced base station and FM TV receiver is mitigated when a TV receiver is placed at angles greater than *θ* = 0°, because antenna radiation gradually moves toward its minimum gain values. However, at arrival angles of *θ* ≥ 92°, the distance becomes constant and shows minimum value, since the inter-system interference is reduced to its minimum powers.

## Conclusion

In this paper, we have proposed a new interference signal power attenuation scheme for coexistence analysis between OFDM-based LTE-Advanced and FM broadcasting. A closed-form for interference power attenuation loss is derived and used to investigate coexistence feasibility at co-channel and various adjacent frequency scenarios. The proposed scheme has computed bandwidth overlapping ratio more exact than other methods and it shows positive and better behavior for large interference power. The proposed model is more beneficial for coexistence feasibility between OFDM-based LTE-Advanced and FM TV services. This model is valid to investigate coexistence situation for any system uses OFDM technology with FM TV system.

## Appendix

The convergence issues are actually come from the closed form series in Eqs (13), (14) and (15) due to summation from *k* = 1 to ∞ and the factorial function. To prove convergence of the closed form series we can use the Test Ratio method of the series in Eqs (13), (14) and (15). This method states that for a given $\sum_{k=1}^{\infty }Q_{k}$, if $\underset{}{{\text{lim}}_{k\to \infty }}\left|\frac{Q_{k+1}}{Q_{k}}\right|=L<1$, then the series is convergent, and if *L* > 1, then the series is divergent. We will prove Eq (13) as an example:

For the first term in the infinite series summation in Eq (13): $\overset{}{\underset{}{\sum_{k=1}^{\infty }\frac{{\left(-1\right)}^{k}\left[{\left(2\pi \left(h_{1}-i\right)\right)}^{2k}-{\left(2\pi \left(h_{2}-i\right)\right)}^{2k}\right]}{\left(2k\right)\left(2k\right)!}}}$ Let $Q_{k}=\frac{{\left(-1\right)}^{k}\left[{\left(x_{1}\right)}^{2k}-{\left(x_{2}\right)}^{2k}\right]}{\left(2k\right)\left(2k\right)!}$, where *x*_1_ = 2*π*(*h*_1_ − 1), and *x*_2_ = 2*π*(*h*_2_ − 1). Then, the infinite series for the first term in in Eq (13) is given as $\sum_{\text{k}=1}^{\infty }Q_{k}.$

$$\underset{k\to \infty }{\text{lim}}\left|\frac{Q_{k+1}}{Q_{k}}\right|=\underset{k\to \infty }{\text{lim}}\left|\frac{\left(2k\right)\left(2k\right)!\left(x_{1}{}^{2k+2}-x_{2}{}^{2k+2}\right)}{\left(2k+2\right)\left(2k+2\right)!\left(x_{1}{}^{2k}-x_{2}{}^{2k}\right)}\right|$$

$$=\underset{k\to \infty }{\text{lim}}\left|\frac{\left(2k\right)\left(x_{1}{}^{2}-{\left(\frac{x_{2}}{x_{1}}\right)}^{2k}x_{2}{}^{2}\right)}{{\left(2k+2\right)}^{2}\left(2k+1\right)\left(1-{\left(\frac{x_{2}}{x_{1}}\right)}^{2k}\right)}\right|$$

Since -1<$\frac{x_{2}}{x_{1}}=\frac{h_{2}-1}{h_{1}-1}$<1, the ${\left(\frac{x_{2}}{x_{1}}\right)}^{2k}=0$, then the above limit of the sequence is rewritten as

$$\underset{k\to \infty }{\text{lim}}\left|\frac{Q_{k+1}}{Q_{k}}\right|=\underset{k\to \infty }{\text{lim}}\left|\frac{\left(2k\right)x_{1}{}^{2}}{{\left(2k+2\right)}^{2}\left(2k+1\right)}\right|$$

$$\underset{k\to \infty }{\text{lim}}\left|\frac{Q_{k+1}}{Q_{k}}\right|=\underset{k\to \infty }{\text{lim}}\left|\frac{\left(2k\right)x_{1}{}^{2}}{k^{3}\left(8+\frac{20}{k}+\frac{16}{k^{2}}+\frac{4}{k^{3}}\right)}\right|$$

=0<1.

Therefore, $\sum_{k=1}^{\infty }Q_{k}$ is convergent.

For the third term in the infinite series summation in Eq (13): $\overset{}{\underset{}{\sum_{k=1}^{\infty }\frac{{\left(-1\right)}^{k-1}\left[{\left(2\pi \left(h_{2}-i\right)\right)}^{2k-1}-{\left(2\pi \left(h_{1}-i\right)\right)}^{2k-1}\right]}{\left(2k-1\right)\left(2k-1\right)!}}}$ Let $Q_{k}=\frac{{\left(-1\right)}^{k-1}\left[{\left(x_{1}\right)}^{2k-1}-{\left(y_{1}\right)}^{2k-1}\right]}{\left(2k-1\right)\left(2k-1\right)!}$, where *x*_1_ = 2*π*(*h*_2_ − 1), *y*_1_ = 2*π*(*h*_1_ − 1). Then, the infinite series for the second term in Eq (13) is given as $\sum_{k=1}^{\infty }Q_{k}$

$$\underset{k\to \infty }{\text{lim}}\left|\frac{Q_{k+1}}{Q_{k}}\right|=\underset{k\to \infty }{\text{lim}}\left|\frac{\left(2k-1\right)\left(2k-1\right)!\left(x_{1}{}^{2k+1}-y_{1}{}^{2k+1}\right)}{\left(2k+1\right)\left(2k+1\right)!\left(x_{1}{}^{2k-1}-y_{1}{}^{2k-1}\right)}\right|$$

$$=\underset{k\to \infty }{\text{lim}}\left|\frac{\left(2k-1\right)}{2k{\left(2k+1\right)}^{2}}\;\frac{\left(x_{1}{}^{2}-{\left(\frac{y_{1}}{x_{1}}\right)}^{2k-1}y_{1}{}^{2}\right)}{1-{\left(\frac{y_{1}}{x_{1}}\right)}^{2k-1}}\right|.$$

Since -1<$\frac{y_{1}}{x_{1}}=\frac{h_{1}-1}{h_{2}-1}$<1, the ${\left(\frac{y_{1}}{x_{1}}\right)}^{2k-1}=0$, and then the above limit of the sequence is rewritten as

$$\underset{k\to \infty }{\text{lim}}\left|\frac{Q_{k+1}}{Q_{k}}\right|=\underset{k\to \infty }{\text{lim}}\left|\frac{\left(2k-1\right)x_{1}{}^{2}}{2k{\left(2k+1\right)}^{2}}\right|$$

$$\underset{k\to \infty }{\text{lim}}\left|\frac{Q_{k+1}}{Q_{k}}\right|=\underset{k\to \infty }{\text{lim}}\left|\frac{k\left(2-\frac{1}{k}\right)x_{1}{}^{2}}{2k^{3}\left(4+\frac{4}{k}+\frac{1}{k^{2}}\right)}\right|$$

$$\underset{k\to \infty }{\text{lim}}\left|\frac{Q_{k+1}}{Q_{k}}\right|=\underset{k\to \infty }{\text{lim}}\left|\frac{\left(2-\frac{1}{k}\right)x_{1}{}^{2}}{2k^{2}\left(4+\frac{4}{k}+\frac{1}{k^{2}}\right)}\right|$$

=0<1.

Therefore $\sum_{k=1}^{\infty }Q_{k}$ is convergent.

## References

[1] WRC-07 resolution 749 [com4/13], “Studies on the use of the band 790–862 MHz by mobile applications and by other services”, 2007.

[2] López-Pérez D, Chu X, Vasilakos AV, Claussen H. On Distributed and Coordinated Resource Allocation for Interference Mitigation in Self-Organizing LTE Networks. IEEE/ACM Transactions on Networking.2013; 21(4): 1145–1158.

[3] Nee R, Prasad R. OFDM for wireless multimedia communications. Artech House, Boston, MA, 2000.

[4] Ye S, Wong SH, Worrall C. Enhanced physical downlink control channel in LTE advanced Release 11. IEEE Communications Magazine. 2013; 51(2): 82–89.

[5] Shamsan ZA, Rahman TA, Al-hetar AM. Point-Point Fixed Wireless and Broadcasting Services Coexistence with IMT-Advanced System. Progress In Electromagnetics Research. 2012; PIER 122: 537–555.

[6] Yang M, Li Y, Jin D, Zeng L, Wu X, Vasilakos AV. Software-Defined and Virtualized Future Mobile and Wireless Networks: A Survey. Mobile Networks and Applications. 2015; 20(1): 4–18.

[7] Youssef M, Ibrahim M, Abdelatif M, Chen L, Vasilakos AV. Routing Metrics of Cognitive Radio Networks: A Survey. IEEE Communications Surveys and Tutorials. 2014; 16(1): 92–109.

[8] He Y, Sun J, Ma X, Vasilakos AV, Yuan R, Gong W. Semi-Random Backoff: Towards Resource Reservation for Channel Access in Wireless LANs. IEEE/ACM Transactions on Networking. 2013; 21(1):204–217.

[9] Shamsan ZA, Rahman TA, Kamarudin MR, Al-hetar AM, Jo H-S. Coexistence of OFDM-Based IMT-Advanced and FM Broadcasting Systems. ETRI Journal. 2011; 33(2): 279–282.

[10] Shamsan ZA, Rahman TA, Al-hetar AM. Interference Coordination for LTE-Advanced and FM Broadcasting Interoperability. Annals of Telecommunications. 2012; 67: 477–483.

[11] Jo H-S, Yoon H-G, Lim J, Chung W-G, Yook J-G, Park H-K. The Coexistence of OFDM-Based Systems beyond 3G with Fixed Service Microwave Systems. Journal of Communications and Networks. 2006; 8(2): 187–193.

[12] Chung W-G, Jo H-S, Yoon H-G, Lim J, Yook J-G, Park H-K. An Advanced MCL Method for a Sharing Analysis of IMT-Advanced Systems. IET Electronics Letters. 2006; 42(21): 1234–1235.

[13] CEPT ERC Report 101, A Comparison Of The Minimum Coupling Loss Method, Enhanced Minimum Coupling Loss Method, And The Monte-Carlo Simulation, May 1999.

[14] Analytical Graphics, Inc. (AGI), Evaluating Communications Links, Available (on 6/8/2016) from: http://www.agi.com/resources/help/online/stk/10.1/index.html?page=source%2Fextfile%2Fcomm%2FCommRadar10-01.htm.

[15] Jo H. S.; Yoon H. G.; Lim J.; Yook J. G., An Advanced MCL Method for Assessing Interference Potential of OFDM-Based Systems beyond 3G with Dynamic Power Allocation, IEEE European Conference on Wireless Technology, 2006, Pages: 39–42, doi: 10.1109/ECWT.2006.280429

[16] Duarte PBF, Fadlullah ZM, Vasilakos AV, Kato N. On the partially overlapped channel assignment on wireless mesh network backbone: A game theoretic approach. IEEE Journal on Selected Areas in Communications. 2012; 30(1): 119–127.

[17] Zhang XM, Zhang Y, Yan F, Vasilakos AV. Interference-Based Topology Control Algorithm for Delay-Constrained Mobile Ad Hoc Networks. IEEE Transactions on Mobile Computing. 2015; 14(4): 742–754.

[18] Wang C-Y, Ko C-H, Wei H-Y, Vasilakos AV. A Voting-Based Femtocell Downlink Cell-Breathing Control Mechanism. IEEE/ACM Transactions on Networking

[19] López-Pérez D, Chu X, Vasilakos AV, Claussen H. Power Minimization Based Resource Allocation for Interference Mitigation in OFDMA Femtocell Networks. IEEE Journal on Selected Areas in Communications. 2014; 32(2): 333–344.

[20] Kroeger B. Adjacent channel interference mitigation for FM digital audio broadcasting receivers. US Patent 7221917, 2007.

[21] De Boer G, Kupferschmidt C, Bederov D, Kuchenbecker H-P. Digital audio broadcasting in the FM band based on continuous phase modulation. IEEE Trans. Broadcasting. 2003; 49(3): 293–303.

[22] BBC response to Ofcom’s Consultation, “TV white spaces: approach to coexistence”, 2013.

[23] ITU-R BT.1895. “Protection criteria for terrestrial broadcasting systems (05/2011)”.

[24] ITU-R Document 3M/TEMP/20-E, DRAFT NEW HANDBOOK on the selection and use of radio propagation prediction methods for interference and sharing studies, 2012.

[25] Spiegel MR. Mathematical handbook of formulas and tables. McGraw-Hill Inc 1968.

[26] Penttinen Jyrki T. J., The Telecommunications Handbook: Engineering Guidelines for Fixed, Mobile and Satellite Systems, page 568, John Wiley & Sons, 2015.

[27] Wijting C, Doppler K, Kalliojärvi K, Svensson T, Sternad M, Auer G, et al Key Technologies for IMT-Advanced Mobile Communication Systems. IEEE Wireless Comm. Mag. 2009; 16(3): 76–85.

[28] 3GPP2/TSG-C.R1002. 1xEV-DV evaluation methodology (V12.1). NOKIA. 2003.

[29] ITU-R P.452-16, (2015). Prediction procedure for the evaluation of interference between stations on the surface of the Earth at frequencies above about 0.1 GHz.

[30] ITU-R Recommendation P. 452–8. Prediction procedure for the evaluation of microwave interference between stations on the surface of the earth at frequencies above about 0.7 GHz. 2005.

[31] ITU-R (Working Party 5D). Liaison Statement to 3GPP and IEEE: Parameters for LTE-Advanced and WirelessMAN Advanced for use in Sharing Studies. Document 5D/TEMP/91(Rev.1). 2012.

